# The Role of 18F-FDG PET/CT in Staging and Prognostication of Mantle Cell Lymphoma: An Italian Multicentric Study

**DOI:** 10.3390/cancers11121831

**Published:** 2019-11-21

**Authors:** Domenico Albano, Riccardo Laudicella, Paola Ferro, Michela Allocca, Elisabetta Abenavoli, Ambra Buschiazzo, Alessia Castellino, Agostino Chiaravalloti, Annarosa Cuccaro, Lea Cuppari, Rexhep Durmo, Laura Evangelista, Viviana Frantellizzi, Sofya Kovalchuk, Flavia Linguanti, Giulia Santo, Matteo Bauckneht, Salvatore Annunziata

**Affiliations:** 1 Nuclear Medicine, University of Brescia, Spedali Civili Brescia, 25123 Brescia, Italy; doalba87@libero.it (D.A.); rexhep.durmo@gmail.com (R.D.); 2 Nuclear Medicine Unit, Department of Biomedical and Dental Sciences and Morpho-Functional Imaging, University of Messina, 98125 Messina, Italy; riclaudi@hotmail.it; 3 Nuclear Medicine Department, IRCCS San Raffaele Hospital, 20132 Milan, Italy; paola.paolina01@gmail.com; 4 Nuclear Medicine Unit, Department of Experimental and Clinical Biomedical Sciences, University of Florence, 50134 Florence, Italy; michimedn2@gmail.com (M.A.); elisabettabenavoli@gmail.com (E.A.); flavialinguanti@hotmail.it (F.L.); 5 Nuclear Medicine Department, S. Croce e Carle Hospital Cuneo, 12100 Cuneo, Italy; ambra.buschiazzo@gmail.com; 6 Hematology Division, S. Croce e Carle Hospital Cuneo, 12100 Cuneo, Italy; castellino.al@ospedale.cuneo.it; 7 Department of Biomedicine and Prevention, University Tor Vergata, 00133 Rome, Italy; agostino.chiaravalloti@gmail.com; 8 IRCCS Neuromed, 86077 Pozzilli, Italy; 9 Istituto di Ematologia, Fondazione Policlinico Universitario A. Gemelli IRCCS, Università Cattolica del Sacro Cuore, 00168 Rome, Italy; annarosa.cuccaro@gmail.com; 10 Nuclear Medicine and Molecular Imaging Unit, Veneto Institute of Oncology IOV-IRCCS, 35128 Padua, Italy; lea.cuppari@aulss2.veneto.it; 11 Nuclear Medicine Unit, Department of Medicine – DIMED, University of Padua, 35121 Padua, Italy; laura.evangelista@unipd.it; 12 Department of Molecular Medicine, Sapienza University of Rome, 00185 Rome, Italy; viviana.frantellizzi@uniroma1.it; 13 Hematology Unit, Department of Experimental and Clinical Biomedical Sciences, University of Florence, 50134 Florence, Italy; sofya.kovalchuk@unifi.it; 14 Nuclear Medicine Unit, Department of Interdisciplinary Medicine, University of Bari Aldo Moro, 70124 Bari, Italy; giuliasanto92@gmail.com; 15 Nuclear Medicine, IRCCS Policlinico San Martino, 16132 Genova, Italy; 16 Institute of Nuclear Medicine, Fondazione Policlinico Universitario A. Gemelli IRCCS, Università Cattolica del Sacro Cuore, 00168 Rome, Italy; salvatoreannunziata@live.it

**Keywords:** mantle cell lymphoma, 18F-FDG PET/CT, prognosis, Deauville criteria

## Abstract

Mantle cell lymphoma (MCL) is an aggressive lymphoma subtype with poor prognosis in which 18F-FDG-PET/CT role in treatment response evaluation and prediction of outcome is still unclear. The aim of this multicentric study was to investigate the role of 18F-FDG-PET/CT in staging MCL and the prognostic role of Deauville criteria (DC) in terms of progression-free survival (PFS) and overall survival (OS). We retrospectively enrolled 229 patients who underwent baseline and end-of-treatment (eot) 18F-FDG-PET/CT after first-line therapy. EotPET/CT scans were visually interpreted according to DC. The sensitivity, specificity, positive predictive value, negative predictive value and accuracy of PET/CT for evaluation of bone marrow (BM) were 27%, 100%, 100%, 48% and 57%, respectively. The sensitivity, specificity, positive predictive value, negative predictive value and accuracy of PET/CT for evaluation of the gastrointestinal (GI) tract were 60%, 99%, 93%, 90% and 91%, respectively. At a median follow-up of 40 months, relapse occurred in 104 cases and death in 49. EotPET/CT results using DC significantly correlated with PFS, not with OS. Instead, considering OS, only MIPI score was significantly correlated. In conclusion, we demonstrated that MCL is an FDG-avid lymphoma and 18F-FDG-PET/CT is a useful tool for staging purpose, showing good specificity for BM and GI evaluation, but suboptimal sensitivity. EotPET/CT result was the only independent significant prognostic factor that correlated with PFS.

## 1. Introduction

Mantle cell lymphoma (MCL) accounts for 3% to 6% of all non-Hodgkin lymphoma and it is an aggressive lymphoma subtype with a high recurrence and mortality rate [1]. In the era of personalized and precision medicine, strong prognostic tools are needed in all lymphoma subtypes, such as MCL, to accurately predict possible further relapse of disease or death and to improve their treatment management. For this end-point, both clinical and imaging parameters are nowadays available, such as clinical scores and fluorine-18-fluorodeoxyglucose positron emission tomography/computed tomography (18F-FDG-PET/CT) derived parameters. MCL International Prognostic Index (MIPI) is the first prognostic index suited for MCL patients and may serve as an important tool to facilitate risk-adapted treatment decisions in patients with advanced stage MCL [2]. At the same time, FDG-PET/CT at the end of first-line therapy is recognized as a powerful prognostic tool in many lymphoma subtypes. The Deauville score criteria (DC) and subsequent Lugano criteria were proposed in this setting and nowadays they are commonly accepted as a tool to evaluate response to treatment and prognosis in both interim and end-of-therapy setting in some lymphoma subtypes as in Hodgkin lymphoma (HL) and large B-cell lymphoma (DLBCL) [3,4]. For other less common lymphoma subtypes such as MCL, there is no clear scientific evidence of the role of these criteria in treatment evaluation and prognostication, although theoretically they can be considered valid. A recent editorial by Bailly et al. [5] underlined the lack of scientific evidences of a proven role of these criteria in MCL. Moreover, 18F-FDG-PET/CT at baseline has been recently evaluated to complete the staging of disease at diagnosis, with possible implication of different prognostic risk in the follow-up. The aim of this study was to evaluate the role of 18F-FDG-PET/CT in patients with MCL as follows: assessing its power in staging MCL; evaluating DC as a predictive tool in response to therapy and as a prognostic tool in terms of progression-free survival (PFS) and overall survival (OS); testing the combination of clinical and imaging parameters in order to identify different risk classes able to predict the outcomes.

## 2. Results

### 2.1. Tumor Characteristics

Among 229 patients with histological proven MCL, there was a prevalence of male (*n* = 172) compared to female (*n* = 57); the average age was 65.1 years (range: 29–88). Patients were staged according to the Ann Arbor system as follows: stage I (*n* = 4), stage II (*n* = 9), stage III (*n* = 32) and stage IV (*n* = 184). B-symptoms, bulky disease and splenomegaly were described in 65, 40 and 101 patients, respectively. LDH level resulted high in 93 cases, β2-microglobulin in 65 and MIPI score was greater than 6 in 130 patients. Proliferative index Ki-67 was available in 183 patients, being low in 65 (36%) and high in 118 (64%) subjects. Baseline features of the patients are summarized in Table 1.

### 2.2. 18F-FDG PET/CT Evaluation for Initial Staging

All patients had abnormal 18F-FDG PET/CT showing the presence of at least one lesion with increased FDG uptake consistent with MCL. 18F-FDG PET/CT scans detected nodal disease in 216 (94%) patients. Splenic involvement was detected by PET/CT in 100 (44%) patients.

Considering bone marrow (BM) biopsy as reference, BM disease was reported in 136 (59%) patients, while 18F-FDG PET/CT showed a focal uptake, compatible with BM involvement, in 37 (16%) subjects. Sensitivity (SE), specificity (SP), positive predictive value (PPV), negative predictive value (NPV) and accuracy (AC) of 18F-FDG-PET/CT in evaluating BM were 27% (20–35%), 100% (96–100%), 100%, 48% (46–51%) and 57% (50–63%); negative likelihood ratio was 0.73. No false positive findings for BM involvement were registered at PET/CT; however, 99 false negative findings were registered (Table 2). Agreement between the two techniques was low (Cohen’s k = 0.23).

18F-FDG PET/CT resulted positive for gastrointestinal (GI) tract involvement in 30 (13%) cases: among them, in 13 showing gastric uptake, 1 in gastro-duodenal junction uptake, 2 in the duodenum, 12 in the colon, 1 in sigma and 1 in the cecum. Considering GI endoscopy as standard of reference, 47 patients had a GI involvement of MCL. SE, SP, PPV, NPV and AC of 18F-FDG-PET/CT were 60% (44–74%), 99% (96–100%), 93% (78–98%), 90% (87–93%), 91% (86–94%) respectively; positive and negative likelihood ratios were 54.21 and 0.41, respectively (Table 2). Agreement between the two techniques was quite good (Cohen’s k = 0.67).

### 2.3. Role of 18F-FDG PET/CT in Predicting Survival

#### Application of Deauville Criteria

**Figure 1:**
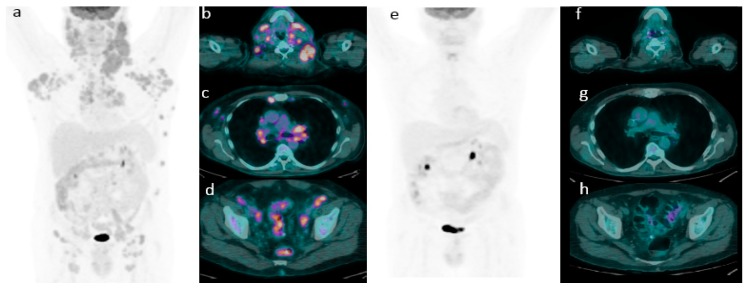
Emblematic example of complete metabolic response. A representative case of a 56-year-old male with stage III MCL. (**a**) Baseline maximum intensity projection (MIP), showing diffuse hypermetabolic disease in (**b**) laterocervical, (**c**) mediastinal and (**d**) iliac nodes. (**e**) PET/CT after chemotherapy showing a complete metabolic response (Deauville score 1) with no 18F-FDG uptake (**f**–**h**) with the disappearance of previous lesions.

**Figure 2:**
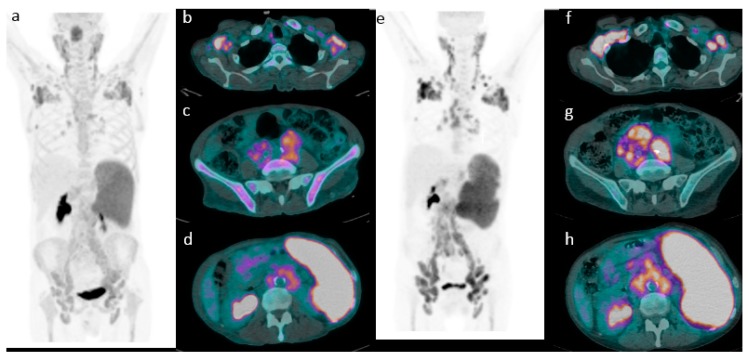
Emblematic example of progressive disease. A representative case of a 65-year-old male with stage III MCL. (**a**) Baseline maximum intensity projection (MIP, showing diffuse hypermetabolic disease in (**b**) laterocervical, axillary, (**c**) iliac and (**d**) inguinal nodes and in spleen. (**e**) PET/CT after chemotherapy showing a metabolic progression of disease (Deauville score 5) with the appearance of new lesions (**f**–**h**).

According to DC, 186 (81%) patients were categorized as eotPET negative (score 1–3) (Figure 1) and 43 (19%) as a positive scan (score 4–5) (Figure 2). At a median follow-up of 40 months, relapse and/or progression of lymphoma was found in 104 patients with a mean time of 31.2 months (range: 3–145) from the diagnosis, and death was found in 49 cases with a mean time of 33.9 months (range 3–145). Considering patients with relapse/progression of disease, a negative eotPET/CT scans applying DC were registered in 74 cases, whilst a positive scan in 30 (in particular, Deauville score 4 in thirteen cases and Deauville score 5 in seventeen). Instead, among patients who died during the course of the disease, eotPET/CT applying DC was positive in 20 (in particular, score 4 in seven cases and score 5 in thirteen) and negative in 27 (in particular, score 1 in 17 subjects, score 2 in nine and score 3 in one). Patients with eot positive PET/CT underwent subsequent second-line chemotherapy regimen according to institutional protocol.

The median PFS and OS were 28 months (range 3–145 months) and 34 months (range 3–145 months) respectively; with estimated 2-year PFS and OS rates of 73% and 84%, and 3-year PFS and OS rates of 56% and 75%, respectively.

**Figure 3:**
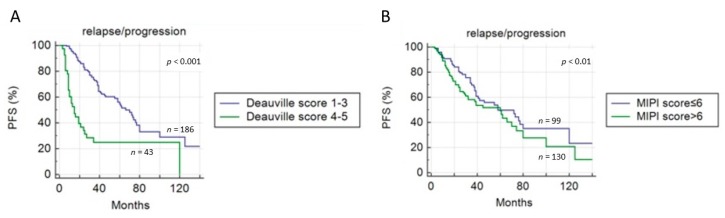
Progression-free survival (PFS) curves according to end-of-treatment PET/CT results using (**A**) Deauville criteria and (**B**) MIPI score.

**Figure 4:**
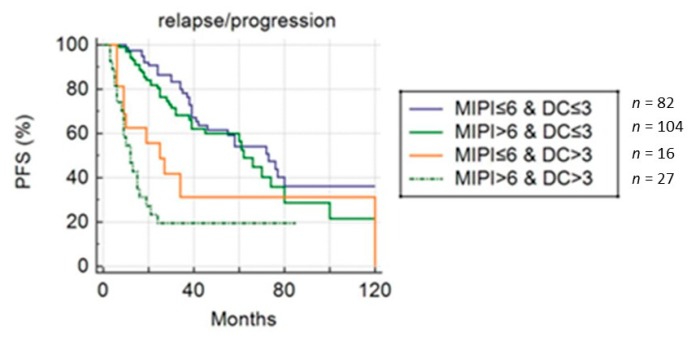
Progression-free survival curve combining MIPI score and Deauville score groups.

At univariate analysis, DC and MIPI score were the only parameters significantly related to PFS (Figure 3); other clinical/pathological features (sex, age, splenomegaly, bulky disease, stage, blastoid variant, β2-microglobulin and LDH level) were not associated with the outcome. PFS was significantly shorter in patients with eotPET/CT DC positive compared to negative (12 vs. 32 months, *p* < 0.001). At multivariate analysis, only metabolic feature (Deauville score) was confirmed to be an independent prognostic factor for PFS (*p* < 0.001). Combining DC (≤3 and >3) and MIPI score (≤6 and >6), PFS was significantly longer in eotPET negative patients independently from MIPI score (*p* < 0.001) (Figure 4). Median PFS was 82 months in patients with low-intermediate MIPI score (≤6) and a negative eotPET/CT (DC ≤ 3); 62 months in patients with high MIPI score (>6) and a negative eotPET/CT; 25 months in patients with low-intermediate MIPI score and a positive eotPET/CT (DC 4 or 5), and 12 months in patients with high MIPI score and a positive eotPET/CT.

**Figure 5:**
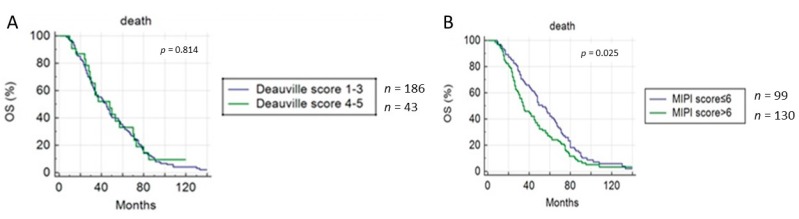
Overall survival (OS) curves according to end-of-treatment PET/CT results using (**A**) Deauville criteria and (**B**) MIPI score.

Instead, considering OS, only MIPI score resulted as significantly correlated (Figure 5), both at univariate and multivariate analysis (*p* = 0.017) (Table 3). EotPET/CT according to DC did not predict OS (31 vs. 37.5 months, *p* = 0.814) (Figure 5).

Treatment regimen (R-CHOP regimen vs. other chemotherapy regimen) did not influence outcome survival considering both PFS and OS.

## 3. Discussion

MCL is an aggressive B-cell non-Hodgkin lymphoma with a fast-growing course, high-risk of relapse and need for early treatment. Patients affected by MCL typically present with advanced-stage disease and extranodal involvement with a predilection for BM and GI tract, where routine conventional techniques such as CT have a suboptimal accuracy [6]. A correct and an early identification of initial and extranodal disease may be crucial because it could potentially affect patient’s management and therapeutic choice. 18F-FDG PET/CT has shown a high accuracy in the detection of nodal involvement [7,8,9], but a low sensitivity in BM and GI tract, being inadequate to replace BM biopsy and GI endoscopy in disease staging [10,11,12]. In the present study, we demonstrated a very low sensitivity of PET/CT in the evaluation of BM involvement (27%), but with a specificity equal to 100%. No case of false-positive findings on BM at PET/CT were recorded, but there were 99 cases of false-negative PET. Thus, the need for BM biopsy seems to be mandatory in case of negative BM PET/CT. Previous studies [10,11,12] reported a very low pretreatment PET scan accuracy to detect BM involvement, very low with sensitivity ranging from 12% to 51%. A comparison between our paper and others is not easy due to the different population number and features evaluated. Recently, Morgan et al. [13] projected a voxel-based analysis of the iliac bones for classifying BM disease in MCL founding a good sensitivity and specificity (100% and 87.5%, respectively); this method seems to be very promising but less practical and reproducible and it has been validated only on a small sample of patients. The potential role of 18F-FDG PET/CT in evaluating lymphoma BM involvement remains a challenge; this is a non-invasive technique well studied in HL and DLBCL, but with controversial and limited evidence [14,15]. Several studies have proven that 18F-FDG PET/CT can be an accurate method for the evaluation of BM involvement in patients affected by DLBCL considering focal highly 18F-FDG-avid lesions as reference standard; on the other hand the absence of a focal uptake does not exclude the presence of BM involvement. Our results confirmed similar evidences also in MCL, suggesting to avoid a BM biopsy in case of positive PET/CT scan.

Also, for the GI tract, our results showed that 18F-FDG PET/CT revealed a good specificity but a low sensitivity (60%), being in agreement with other published papers [9,12,16]. It was already demonstrated that an increased 18F-FDG bowel uptake may be common, especially in diabetic patients [17,18], thus reducing the diagnostic accuracy of the evaluation of this district.

Another crucial topic to consider in MCL is the prognostication; nowadays, neither clinical nor imaging prognostic markers are available in MCL setting. MIPI score [2] was an attempt created to better classify patients with MCL and predict prognosis, but its validation has shown conflicting results [19,20]. Other histopathological features, such as blastoid variant and Ki-67 score, have been investigated without shared evidence [21]. Instead, the prognostic value of 18F-FDG-PET/CT in MCL remains under debate [16,22,23,24,25]. In 2009, a five-point scale system called Deauville criteria (DC) was introduced to analyze 18F-FDG PET/CT results after treatment in lymphoma [26]; this scale used the mediastinum and liver activity as the reference standard, and has been recommended for reporting both interim and end-of-treatment PET for FDG-avid lymphoma (both HL and NHL, especially DLBCL) [3,4,27]. In our paper, we tried to validate this score also in MCL, obtaining some strong results; patients with a negative end-of-treatment PET/CT (DC1-3) had significantly longer PFS than patients with a positive PET/CT (DC4,5). Similar results in small cohort of patients were reported by other authors [20,22,28]. However, this evidence was not confirmed for OS, where DC seems to not influence the outcome survival. This could be explained to the fact that eotPET/CT results were used to guide subsequent management and further treatments, as second-line chemotherapy regimen.

The treatment of MCL is a challenge and often stays unsatisfactory; complete responses to the standard chemotherapy regimens are not common. Czuczman et al. [29] in a multicenter open-label single-arm phase II study showed that the complete metabolic response at PET/CT was a predictor of outcome survival. In addition to qualitative 18F-FDG PET/CT analysis using DC, also semiquantitative PET/CT parameters have been studied as prognostic factors, like SUVmax, with controversial results. Karam et al. [30] and Bodet-Milin et al. [16], in patients affected by MCL, suggested SUVmax thresholds of 5 and 6, respectively, to stratify patients into high-risk versus low-risk groups and to predict OS and disease-free survival. On the other hand, no prognostic impact of SUVmax was demonstrated by other groups [10,12,24]. Recently in the LyMa-PET Project [23], it was demonstrated that SUVmax was the only independent prognostic factor for PFS and OS; moreover, authors suggested a scoring system combining MIPI and SUVmax (using a cutoff of 10.3) to help the outcome prediction. In our study, eotPET/CT results using DC were superior than the MIPI score in PFS, while for OS only the MIPI score resulted as an independent prognostic factor.

Instead, no clinical or histological variables, like blastoid variant and Β2 microglobulin, showed significant association with outcome survival in our analysis; this is partially in contradiction with literature [1] and related probably to heterogeneity of population studied.

Also, Ki-67 score seems not to be related to PET/CT features and outcome survival despite that in other lymphoma subtypes significant correlation was demonstrated [31,32].

The present study has some limitations. First, this is a retrospective multicentric study, so heterogeneous features in our population in terms of clinical baseline parameters and treatment schemes could be found. Despite this, so far and to best of our knowledge, the present study is the first large series of MCL patients aimed to assess the diagnostic and prognostic role of 18F-FDG PET/CT. Second, although this study showed the independent prognostic value for PFS of end-of-treatment PET/CT, the role of semi-quantitative PET/CT parameters, in terms of SUVs, should be further analyzed. Third, more complex statistical analysis, like a deep learning approach, might have improved data analysis.

## 4. Materials and Methods

### 4.1. Patients

Between January 2007 and December 2018 in nine Italian Nuclear Medicine Centers, 229 patients with newly diagnosed, histologically proven MCL were retrospectively enrolled. The histopathological diagnosis was based on the World Health Organization criteria [33]. The inclusion criteria were as follows: patients with a histological confirmation of MCL; age ≥18 years; availability of BM biopsy and GI endoscopy at time of diagnosis; availability of baseline and end of treatment 18F-FDG PET/CT results in all patients; and availability of clinical/radiologic follow-up data for at least 12 months. Patients with concomitant malignancies were excluded. We further reviewed the follow medical records of these patients: epidemiological features (age at diagnosis, sex), clinical data (tumor stage, MIPI score, bulky disease, splenomegaly, B symptoms, lactate-dehydrogenase (LDH) level, β2-microglobulin level), histopathological data (Ki-67 score, blastoid variant), metabolic features by 18F-FDG PET/CT, treatment modalities and follow-up data. LDH level, β2-microglobulin and MIPI score were dichotomized using cutoff values of 245 U/L, 2.8 mg/L and 6 respectively. Tumor stage according to Ann Arbor classification was divided in early (I and II) and advanced (III and IV) disease. The bulky disease was defined when the maximum width was equal or greater than one-third of the internal transverse diameter of the thorax or at an alternative site as any mass measuring 10 cm or more by any imaging study. Proliferative activity, expressed by Ki-67 score, was available in 183 patients; average Ki-67 was 25% (range 1–95%). The Ki-67 expression level was arbitrarily dichotomized using a cut-off of 15% as suggested by other authors [24,34].

All patients were treated according to the institution’s standard protocol with chemotherapy regimen related to the stage of disease, age and institutional internal protocol. Eighty-two patients were treated according to R-CHOP (Rituximab, Cyclophoshpamide, Hydroxydaunomycin, Oncovin, Prednisone) regimen, sixty-eight with R-DHAP (dexamethasone, cytarabine, cisplatin), thirty-nine with R-Bendamustina, twenty-four with BCVPP (Carmustine, Cyclophosphamide, Vinblastine, Procarbazine, and Prednisone), twelve with MBVD (Myocet+BVD). In one case, chemotherapy was followed by the involved field radiotherapy. Four patients (2%) underwent only radiotherapy due to the early stage of disease (stage I).

### 4.2. 18F-FDG PET/CT Imaging and Interpretation

All patients underwent baseline 18F-FDG PET/CT and a subsequent PET/CT at completion of first-line therapy (eotPET/CT). PET/CT studies were performed following EANM guidelines [35]. Baseline PET/CT was performed within 14 days before the first cycle of chemotherapy and end-of-treatment PET/CT was done at least three weeks after the completion of chemotherapy or 12 weeks after completion of radiotherapy.

18F-FDG-PET/CT was performed after at least 6 h of fasting and with glucose level lower than 150 mg/dL. An activity of 3.5–4.5 MBq/Kg of 18F-FDG was administered intravenously and scans were acquired about 60 min after injection, usually from the skull basis to the mid-thigh. Owing to the multicenter nature of the study, the exams were acquired on different PET/CT scanners: Discovery STE or 690 (GE Healthcare Technologies, Milwaukee, Wisconsin, USA), Discovery IQ (GE Healthcare Technologies), Discovery LS (GE Healthcare Technologies), Gemini TFEGXL (Philips Medical Systems, Cleveland, OH, USA) and Biograph 16 (Siemens Healthcare, Erlangen, Germany). Standard parameters used were: CT: 80 mA, 120 Kv without contrast; 2.5–4 min per bed-PET-step of 15 cm; the reconstruction was performed in a 128 × 128 matrix and with a 60 cm field-of-view.

Patients were instructed to void before imaging acquisition, no oral or intravenous contrast agents were administrated and no bowel preparation was used for any patient; written consent was obtained before studies. The PET images were visually analyzed and readers had knowledge of clinical history. PET/CT scans were interpreted visually by two nuclear medicine physicians (DA, SA) with experience in this field and the discordance was resolved by a consensus between the two imaging readers.

For the interpretation of images, each focal tracer uptake deviating from physiological distribution and background and uptake higher than liver activity was considered as suggestive for lymphoma.

In the evaluation of BM involvement, PET/CT findings were considered positive in the presence of isolated/multiple focal uptakes in the BM that could not be explained by benign findings on the underlying CT scan or patient’s medical history (like fractures, spondylodiscitis, etc.). In the evaluation of GI involvement, PET/CT findings were considered positive in the presence of isolated/multiple intense focal uptakes in the GI tract. Accuracy of 18F-FDG PET/T results was evaluated considering further evaluations: lesions with 18F-FDG PET/CT uptakes were considered as true-positives if further analyses confirmed the malignant nature, and false-positive if further evaluations showed no malignant lesions. Lesions without 18F-FDG PET/CT uptakes were considered as true negative if further analyses confirmed the absence of neoplastic lesions and false-negative if further evaluations showed malignant ones. Eot 18F-FDG PET/CT was interpreted by visual analysis using the Deauville 5-point scale [4]. According to Deauville Criteria (DC), 18F-FDG PET was interpreted as follows: 1 = no uptake above background, 2 = uptake equal to or lower than mediastinum, 3 = uptake higher than mediastinum and lower than liver, 4 = uptake moderately increased compared to the liver and 5 = uptake markedly increased compared to the liver. Eot 18F-FDG PET/CT scans were considered negative for scores 1 to 3 and positive for scores 4 to 5.

### 4.3. Statistical Analysis

Our statistical analysis was carried out using MedCalc Software version 17.1 for Windows (Ostend, Belgium). Categorical variables were described as the calculation of simple and relative frequencies, while the numeric variables were described as average, minimum and maximum. We calculated SE, SP, PPV, NPV and AC based on Bayes’s law, with 95% confidence intervals, considering final diagnosis as a reference.

The PFS was calculated from the date of diagnosis of MCL to the date of first relapse, progression of disease or the last follow-up date. The OS was calculated from the date of diagnosis of MCL to the date of death from any cause or the last follow-up date.

Survival curves were plotted according to the Kaplan–Meier method and differences between several groups were investigated by using a two-tailed log rank test. Cox regression was used to evaluate the hazard ratio (HR) and its confidence interval (CI). A *p*-value of <0.05 was considered statistically significant and the adjusted significant *p*-value after Bonferroni’s correction was 0.038.

## 5. Conclusions

In conclusion, in this study, we demonstrated that MCL is an FDG-avid lymphoma and that 18F-FDG PET/CT is a useful tool for staging purpose, which should be considered in the diagnostic flow-chart of this patients in concert with other modalities.

PET/CT showed good specificity for BM and GI evaluation but suboptimal sensitivity. Moreover, eotPET post-therapy PET/CT results, evaluated according to Deauville criteria, can be considered powerful prognostic information in order to stratify the progressive patients.

## Figures and Tables

**Table 1:** Baseline features of our population.

Variables		Patients *n* (%)	Average (Range)
Age (years)			65.1 (29–88)
Sex			
	male	172 (75%)	
	female	57 (25%)	
Tumor stage at diagnosis (Ann Arbor)			
	I	4 (2%)	
	II	9 (4%)	
	III	32 (14%)	
	IV	184 (80%)	
Blastoid variant		26 (11%)	
B symptoms		65 (28%)	
LDH			
	≤245 U/L	121 (57%)	
	>245 U/L	93 (43%)	
β2-microglobulin			
	≤2.8 mg/L	108 (62%)	
	>2.8 mg/L	65 (38%)	
MIPI score			
	low-intermediate (≤6)	99 (43%)	
	high-intermediate (>6)	130 (57%)	
Bulky disease		40 (17%)	
Splenomegaly		101 (44%)	
Ki-67 score			
	≤15%	65 (36%)	
	>15%	118 (64%)	

LDH: lactate dehydrogenase; MIPI: mantle cell lymphoma international prognostic index.

**Table 2:** Agreement between 18F-FDG PET/CT and BM biopsy and GI endoscopy findings.

PET/CT Findings	BM Biopsy		GI Endoscopy	
	Positive	Negative	Positive	Negative
Positive	37 (16%)	0 (0%)	28 (12%)	2 (1%)
Negative	99 (43%)	93 (41%)	19 (8%)	180 (79%)
Total	136 (59%)	93 (41%)	47 (21%)	182 (79%)

BM: bone marrow; GI: gastrointestinal.

**Table 3:** Univariate and multivariate analyses for PFS and OS.

Variables	Univariate Analysis		Multivariate Analysis	
	*p*-Value	HR (95% CI)	*p*-Value	HR (95% CI)
PFS				
Sex	0.451	0.845 (0.548–1.306)		
Age	0.153	1.530 (0.838–3.097)		
MIPI score	0.009	0.713 (0.482–1.056)	0.174	1.219 (0.915–1.623)
LDH level	0.163	0.742 (0.488–1.128)		
Β2 microglobulin	0.458	0.831 (0.511–1.353)		
Ki-67 score	0.066	0.653 (0.415–1.028)		
Bulky disease	0.153	1.722 (0.992–2.987)		
Splenomegaly	0.087	0.703 (0.472–01049)		
Stage acc Ann Arbor	0.855	0.957 (0.589–1.531)		
Blastoid variant	0.185	0.598 (0.282–1.270)		
Deauville score	<0.001	0.137 (0.073–0.259)	<0.001	4.059 (2.573–6.403)
Treatment regimen	0.655	0.857 (0.519–1.243)		
OS				
Sex	0.211	1.759 (0.577–6.033)		
Age	0.375	1.270 (0.722–2.369)		
MIPI score	0.025	0.711 (0.527–0.959)	0.017	1.204 (1.032–1.403)
LDH level	0.709	0.942 (0.690–1.287)		
Β2 microglobulin	0.524	1.128 (0.778–1.635)		
Ki67 score	0.195	1.250 (0.891–1.754)		
Bulky disease	0.390	0.828 (0.539–1.272)		
Splenomegaly	0.287	0.846 (0.622–1.150)		
Stage acc Ann Arbor	0.393	0.859 (0.606–1.217)		
Blastoid variant	0.075	0.618 (0.363–1.051)		
Deauville score	0.814	1.055 (0.671–1.660)		
Treatment regimen	0.598	1.001 (0.571–1.460)		

PFS: progression-free survival; OS: overall survival; HR: hazard ratio; CI: confidence interval; N: number.
